# An exploration and analysis of the role positioning, duty comparison, and career development path of clinical research nurses in developed and developing countries

**DOI:** 10.3389/fmed.2025.1708032

**Published:** 2026-01-05

**Authors:** Xiaotao Cao, Jiaxing Xu, Mingyang Li, Li Zheng

**Affiliations:** 1 Department of Clinical Trial Center, West China Hospital, Sichuan University, Chengdu, Sichuan, China; 2 West China School of Nursing, Sichuan University, Chengdu, Sichuan, China; 3 West China School of Medicine, Sichuan University, Chengdu, Sichuan, China; 4 Department of Orthopedic Surgery and Orthopedic Research Institute, West China Hospital, Sichuan University, Chengdu, Sichuan, China; 5 National Medical Products Administration Key Laboratory for Clinical Research and Evaluation of Innovative Drugs, Clinical Trial Center, West China Hospital, Sichuan University, Chengdu, Sichuan, China

**Keywords:** clinical research nurses, role positioning, duty comparison, career development path, developed and developing countries

## Abstract

With the standardization of the clinical trial industry, clinical research nurses, as core members of multidisciplinary research teams, their role positioning, duties, and career development paths have become critical factors in improving the quality of clinical trials. This review compares and analyses the current practices of clinical research nurses in developed and developing countries, taking China as an example. It sorts out differences in their role perceptions, duty divisions, career promotion paths, and analyses the obstacles affecting the career development of clinical research nurses in China. It aims to provide a useful reference for the professional construction of clinical research nurses in China and other developing countries, help optimize the cultivation mode and management mechanism of clinical research nurses, and then promote the high-quality development of the clinical trial industry in developing countries.

## 1 Introduction

### 1.1 Research background and significance

Against the background of the rapid development of global pharmaceutical R&D and medical research, clinical trials, as a key link in the transformation of medical innovation, have been rising in scale and complexity. According to the statistics, in recent years, the number of clinical trials worldwide has been growing at a rate of about 10% per year (1), involving an increasingly wide range of disease fields, from common chronic diseases to rare diseases, from traditional drug trials to emerging gene therapy and cell therapy trials. This kind of expansion not only brings the hope of more medical breakthroughs, but also puts forward refined and professional requirements for the management and execution of clinical trials.

As a core member of the clinical trial team, the Clinical Research Nurse (CRN) plays an irreplaceable role in the recruitment and management of study participants, coordination of the trial process, data collection and quality control, and has gradually become an important professional force to ensure the smooth progress of clinical trials. Research has shown that increasing recognition and consultation of research nurses, enhancing their professional status within organizations, can help maximize their contributions to healthcare and patient care (2).

Compared with other countries that carried out the professional construction of CRNs earlier, China’s clinical research nurse position started relatively late. In 2012, Shanghai University of Traditional Chinese Medicine Affiliated Shuguang Hospital established the first full-time clinical research nurse position in China, marking the beginning of the exploration of the professionalization of clinical research nursing in China. Since then, although some large tertiary hospitals and clinical trial institutions have gradually introduced clinical research nurses, the overall situation is still in the primary stage of development. At present, clinical research nurses in China generally face the dilemma of ambiguous role positioning, and in practice, there are often cases of unclear intersection of duties with clinical nurses, clinical research coordinators (CRCs), and other roles, which leads to unclear work content and low work efficiency. Due to the lack of unified professional standards and norms, there are also many obstacles in the career development path of clinical research nurses. Problems such as poor promotion channels and an incomplete continuing education system have limited the improvement of professional competence and career development opportunities, thereby affecting the overall quality improvement of clinical trials in China. Therefore, an in-depth discussion of the role definition, duty comparison, and career development path of clinical research nurses in developed and developing countries is of great practical significance for clarifying the professional direction of clinical research nurses in China, improving the construction of the professional system, and promoting the quality of clinical trials.

### 1.2 Overview of the current status of research in developed and developing countries

The development of clinical research nurses in developed countries is relatively mature, especially in the US, the UK, and other countries, and has formed a well-standardized role definition and career system. The National Institutes of Health (NIH) clearly defines clinical research nurses as registered nurses involved in clinical research nursing practice, who assume multiple duties in clinical trials, such as nursing care of study participants, research coordination, and data management (3). The US has also established the International Association of Clinical Research Nurses (IACRN) and has developed professional industry standards and training courses that provide strong support for the career development of clinical research nurses. In the UK, a clinical research nurse is defined as a nurse who works primarily in research in a clinical setting, and their career development path is clear, from junior research nurses to senior research nurses to research nursing specialists, with clear competency requirements and promotion criteria at each stage, and continuing education programs are conducted in cooperation with colleges and universities and scientific research institutions to continuously improve the professional quality of clinical research nurses. Research shows that it is feasible for clinical nurses to participate in clinical research, which can enhance their understanding of the research process and promote collaboration between researchers and clinical personnel. Research-related procedures need to be integrated into the daily work functions of clinical nurses. The participation of anesthesia nurses in research design can greatly improve the research process (as they have an in-depth understanding of the patient’s rehabilitation process) (4).

On the contrary, in most developing countries, for example, China, clinical research nurses are still in the stage of exploration and development. At present, the definition of clinical research nurses in China has not been completely unified, and in most cases, the role of clinical research nurses is to some extent dependent on clinical nurses or CRCs. In terms of job duties, there is a lack of standardized operating processes and a clear definition of duties, leading to large differences in the job content of clinical research nurses in different regions and institutions. In terms of career development, although some hospitals have begun to pay attention to the cultivation of clinical research nurses and try to establish a training system and promotion mechanism, overall, there is a lack of systematization and standardization, and the sense of professional identity and belonging of clinical research nurses is relatively low. Research on clinical research nurses in China mostly focuses on status quo investigation, role exploration. A qualitative study on the ideal professional competence profile of Chinese CRN shows that there are four main competencies that CRN should possess: theoretical knowledge, practical skills, professional qualities, and personality traits. This provides a fundamental framework for enhancing CRN training and career development in China (1). However, further in-depth research and practical exploration are still needed on how to build a career development path for clinical research nurses that meets China’s national conditions (5).

## 2 Comparison of the role positioning of clinical research nurses in developed and developing countries

### 2.1 Role positioning of clinical research nurses in developed countries

#### 2.1.1 Core role definition

There is a clear and mature definition system for the role positioning of clinical research nurses in developed countries. NIH defines clinical research nurses as registered nurses with Good Clinical Practice (GCP) certification, whose core tasks are to focus on the accurate execution of study protocols, provide comprehensive care for study participants, and effectively protect the rights and interests of study participants. In the Phase I trial of the novel coronavirus vaccine, the clinical research nurses took on the important duty of assisting the study participants in signing the informed consent form, explaining in detail to the study participants the purpose of the trial, the process, the potential risks and benefits of the trial, to ensure that the study participants made the decision to participate on their own on the basis of a full understanding of the trial. At the same time, the nurses also closely monitored the various reactions of the study participants after vaccination and promptly identified and documented adverse events (AEs), which provided key data for the safety assessment of the vaccine.

The increasing complexity of clinical trials requires a fully integrated multidisciplinary team. Interaction and communication strategies between nurses and CRCs are critical to optimizing the implementation of clinical trials (6). The Association of Clinical Research Professionals (ACRP) in the UK, on the other hand, sees clinical research nurses as core members of a multidisciplinary team, emphasizing their key role in research coordination, data management, and cross-departmental communication. In a large-scale clinical trial on cardiovascular disease, clinical research nurses actively coordinated with different professionals, such as doctors, pharmacists, and statisticians, to ensure that all parties agreed on the research objectives and operational processes. They were responsible for collecting, organizing, and entering the clinical data of the study participants, using their professional knowledge to carry out a preliminary review of the data to ensure the accuracy and completeness of the data, and laying a solid foundation for the subsequent data analysis and conclusions of the research (7).

#### 2.1.2 Role scope

##### 2.1.2.1 Assistant of researchers

Clinical research nurses in developed countries are deeply involved in the trial protocol design process, and with their rich clinical experience and professional knowledge, they conduct a comprehensive assessment of the feasibility of the trial from the nursing perspective. During the trial, they keep close contact with researchers and monitors (3). In the Phase I trial of antitumor drugs, nurses will pay close attention to the safety issues during the dose escalation, combine the physical condition of the study participants, drug reactions, and other factors, provide timely feedback to the research team on the potential risks, and assist in the adjustment of the trial protocol to ensure the smooth progress of the trial.

##### 2.1.2.2 Protector of study participants

Clinical research nurses lead the informed consent process, adopt easy-to-understand language and diversified communication methods to explain the various aspects of the trial to the study participants, respect the autonomy of the study participants, and ensure that they voluntarily participate in the trial after fully understanding the trial information. In the bioequivalence trial, the research nurses will not only carefully design diary cards to help the study participants accurately record their daily medication intake and physical reactions, but also provide detailed training to the study participants to enable them to master the correct recording methods, effectively protect the privacy of the study participants, and ensure the reliability of the trial data. In addition, for example, in oncology clinical research, clinical research nurses ensure the safety of study participants throughout the process by monitoring toxicity and nursing interventions, and provide ongoing education and psychological support to study participants (8).

##### 2.1.2.3 Controller of the quality

Clinical research nurses shoulder the important mission of monitoring protocol adherence, and strictly follow the trial protocol and GCP requirements to supervise the entire research process. Once a protocol violation is found, they will promptly report this to the researcher and assist in analyzing the reasons and proposing corrective measures. Since the construction of the phase I clinical ward with oncology specialty characteristics in Hunan Provincial Tumor Hospital, through the strict monitoring by the clinical research nurses, the rate of protocol violation in the phase I ward is less than 1%, the incidence of out-of-window blood collection and biological sample handling is below 0.8%, and the incidence of AEs in subjects’ care is less than 0.45%, which greatly improves the quality of the trial (9).

### 2.2 Role positioning of clinical research nurses in China

#### 2.2.1 Localized role characteristics

In China, the role of clinical research nurses has obvious localized characteristics. At present, the more blurred role boundaries are a prominent issue, and most clinical research nurses are part-time clinical nurses, which leads to their duties often overlapping with CRCs in practice. Due to the lack of systematic professional training and clear role definition, many clinical research nurses have failed to obtain independent professional identities, making it difficult to fully realize their professional value in clinical trials. According to the survey, in 80% of tertiary hospitals, clinical research nurses have not received systematic GCP training, which often makes them overwhelmed when facing complex clinical trial requirements (10).

In terms of core functions, the focus of clinical research nurses in China is mainly at the executive level, with basic work such as study participant care and data recording. In practice, nurses’ participation in scientific research activities is insufficient, and although they realize the value of scientific research, they rarely integrate research methods into clinical practice (11). They have fewer opportunities to participate in the research design and decision-making process, which, to some extent, limits the comprehensive development of their professional competencies. Only 35% of the clinical research nurses have participated in the trial protocol review, and their participation in the research decision-making process is low, which prevents them from giving full play to their professional strengths and providing more in-depth and broader support for the clinical trials (12).

#### 2.2.2 Distinction from related positions

##### 2.2.2.1 Vs. clinical nurses

Clinical research nurses differ significantly from clinical nurses in their focus. Clinical research nurses focus on specialized nursing care in research scenarios, for example, in phase I wards, special nursing care, like electric pulse technique operations, may be involved. These operations are closely related to the research objective of clinical trials and require nurses to have specific, specialized skills and knowledge. Clinical nurses, on the other hand, focus on routine clinical care. In a study exploring orthopaedic nurses’ perceptions of research priorities and perceived barriers to participating in research, nurses generally believed that research was not part of their job and that they lacked the relevant knowledge, skills, support, and autonomy (13). Instead, they are mainly responsible for patients’ daily nursing care, condition observation, and basic treatment, in order to meet the basic medical care needs of patients.

##### 2.2.2.2 Vs. CRC

Clinical research nurses differ from CRCs in terms of qualifications and job content. Clinical research nurses must have the qualification of registered nurses, which enables them to perform various nursing operations, such as blood collection, medication administration, and directly provide medical care services for study participants. CRCs, on the other hand, are mostly from non-nursing backgrounds, and their work focuses on administrative coordination, mainly responsible for communication and liaison with all parties, organizations, and filing of documents and materials, and coordination and arrangement of the trial process, in order to ensure the smooth progress of the clinical trial (1).

Through the comparison chart in Table 1, it can be clearly seen that clinical research nurses in developed countries are clearer and more professional in their role positioning, covering multiple key aspects from research design to execution, and playing a core role in the multidisciplinary team. While clinical research nurses in developing countries, although assuming important duties in aspects such as study participants care, still have some opportunities for improvement in terms of role independence, research participation and collaborative division of labor with related positions, and need to further clarify their role positioning and enhance their professional competence in order to adapt to the needs of the continuous development of the clinical trial industry.

**Table 1:** Differences in the role positioning of clinical research nurses in developed and developing countries.

Comparison item	Clinical research nurses in developed countries	Clinical research nurses in developing countries
Core role definition	Registered nurses with GCP certification, focusing on study protocol execution, study participants’ care and protection of rights and interests (NIH definition); core members of multidisciplinary teams, responsible for research coordination, data management, and cross-departmental communication (ACRP definition).	Blurred role boundaries, mostly part-time clinical nurses, lack of independent professional identities; core functions focused on execution, with a focus on study participants’ care and data recording.
Role scope—assistance of researchers	Involved in trial protocol design and feasibility assessment.	Less involved in research design and decision-making, with low involvement in trial protocol review (~35%).
Role scope—protector of study participants	Leads the informed consent process and ensures participant autonomy and privacy.	Involved in the informed consent process, but needs to improve in terms of dominance and professionalism.
Role scope—controller of the quality	Monitors protocol adherence and reports protocol violations in a timely manner.	Monitor protocol adherence, but are relatively weak in detecting and dealing with protocol violations.
Distinction from clinical nurses	Focuses on specialized nursing care in research scenarios, e.g., electric pulsing in phase I wards.	Focus on routine clinical care.
Distinction from CRC	Requires RN qualification to perform nursing operations (blood collection, medication administration).	Mostly non-nursing background, focusing on administrative coordination.

## 3 Comparison of the duties of clinical research nurses in developed and developing countries

### 3.1 CRN duty system in developed countries

#### 3.1.1 Management of the whole trial cycle

Studies have shown that nurse-led research is critical to improving patient care and advancing nursing science (14). Clinical research nurses in developed countries assume all-around and refined duties in the management of the whole trial cycle. Before the trial, nurses need to receive relevant education to enhance their understanding and knowledge level of the clinical trial requirements (15). In the preliminary stage, clinical research nurses are deeply involved in the preparation of ethical review materials, and with their deep understanding of study protocols and clinical practice, they provide professional nursing perspectives and risk assessment to ensure that the ethical review passes smoothly. When developing study participant recruitment strategies, they actively innovate and adopt diverse approaches to attract potential study participants. For example, they enhance enrollment efficiency by holding patient education sessions to introduce the purpose, process, and expected benefits of the trial to patients in detail, answer their questions, and eliminate their concerns. According to the statistics, in a clinical trial on cardiovascular disease, effective patient education sessions resulted in a 30% increase in the recruitment speed of study participants and a significant improvement in the quality of enrollment (16).

In the mid-term of the trial, the clinical research nurses strictly execute the dosing regimen and accurately control every step of the process. Taking the phase I trial of antitumor drugs as an example, during the gradient dose infusion process, the nurses closely monitor the vital signs and drug reactions of the study participants, and timely adjust the infusion speed and dose according to the individual differences of the study participants to ensure the safety and effectiveness of drug therapy. At the same time, they record AEs and serious adverse events (SAEs) in real time, and once an abnormal condition occurs, they immediately activate the emergency response plan and take effective rescue measures to protect the life safety of the study participants (1).

In the late stage of the trial, clinical research nurses lead the follow-up work of study participants and establish a perfect follow-up system. Taking nurses’ long-term follow-up after hematopoietic cell transplantation (HCT) as an example, the nurses contact the study participants on a regular basis to find out their physical conditions, collect information on adverse reactions, and analyze and summarize the data. They also assist in data cleaning and summary report writing, and use their professional knowledge to review and validate the data to ensure the accuracy and reliability of the data, providing strong support for the presentation of the research results (17).

These duties of research nurses are crucial for ensuring the accuracy and completeness of clinical trials (18).

#### 3.1.2 Specialized scope of operation

##### 3.1.2.1 Special techniques

Clinical research nurses in developed countries are skilled in all kinds of research-specific skills to provide technical guarantees for the smooth execution of clinical trials. In studies involving the operation of electric pulse equipment, nurses are professionally trained to accurately operate the equipment to provide safe and effective treatment for study participants. In terms of cold chain management of biological samples, they strictly control the temperature of sample collection, transport, and storage to ensure that the quality and activity of biological samples are not affected, providing reliable samples for subsequent laboratory testing and analysis (19, 20).

##### 3.1.2.2 Interdisciplinary collaboration

Clinical research nurses actively collaborate with pharmacologists, statisticians, and other interdisciplinary team members to jointly advance clinical trials. In pharmacokinetic studies, nurses cooperate closely with pharmacologists to accurately collect pharmacokinetic specimens according to the study protocol and strictly control the collection time window to ensure that the collected specimens accurately reflect the metabolic process of drugs in the body. They also cooperated with statisticians to participate in data collection and analysis, using their professional knowledge to ensure that data collection met the requirements of the protocol, providing high-quality data for statistical analysis and helping to ensure the accuracy and reliability of the research conclusions (14). In a study of the unique role of the pediatric clinical research nurse, CRNs were required to collaborate with healthcare professionals in multiple departments, including preoperative, intraoperative, and postoperative phases. The number of collaborating departments has increased by 750% over the past 7 years (21, 22).

### 3.2 Current status of CRN duties in China

#### 3.2.1 Focus of core duties

The core duties of clinical research nurses in China mainly focus on the basic execution level. They are primarily responsible for coordinating and managing clinical trial programs and subject treatment in accordance with study protocols (23). In the study participant screening process, nurses seriously check the entry criteria and ensure that the selected study participants meet the research requirements through meticulous enquiry and examination. In a diabetes drug clinical trial, nurses conducted detailed medical history enquiries and blood glucose tests on each potential study participant, and strictly followed the entry criteria to ensure the homogeneity of the study population.

Clinical research nurses play an important role in bridging the gap during the informed consent process. They translate technical terms into plain language and explain in detail the content, risks, and benefits of the trial to the study participants to ensure that they fully understand and sign the informed consent form voluntarily. In the process of orthopaedic treatment, nurses help study participants understand the complex principles through vivid analogies and patient explanations, enabling them to make well-informed decisions about participation (13).

Clinical research nurses are also responsible for the distribution and recovery of trial drugs, distributing drugs in strict accordance with prescribed procedures and dosages, and recovering the remaining drugs in a timely manner to ensure the safe management of the drugs. During the drug distribution process, they carefully check the study participant information and drug information to ensure accuracy. Meanwhile, the recovered drugs are properly disposed of to prevent drug abuse and waste.

In terms of data management, CRNs are responsible for collating and managing all documents and files from clinical trials to ensure data completeness and accuracy (24). Clinical research nurses in China are mainly responsible for manually entering case report forms (CRFs) and organizing trial documents. However, this way of working is less efficient and prone to errors. According to the survey, 90% of clinical research nurses need to spend 40% of their working time dealing with paper documents, which not only consumes a lot of human and material resources but also increases the risk of data entry errors, which affects the progress of the clinical trial and data quality (23).

#### 3.2.2 Gap in fulfilling duties

At present, there is a gap between clinical research nurses in China and developed countries in terms of duty fulfilment. In terms of research participation, 67% of nurses have never participated in nursing research, which makes it difficult for them to take on research responsibilities after entering the workforce (25). For example, due to the lack of in-depth participation in research design, the violation rate of trial protocols in China is much higher than the international standard. The lack of participation of clinical research nurses in the design of the trial process and the loopholes in the assessment and monitoring of the study participants also led to frequent protocol violations, which affected the smooth progress of the trial and the reliability of the research results.

Clinical research nurses in China are also relatively weak in terms of ethical review capability. Less than 20 per cent of clinical research nurses were able to independently identify potential ethical risks (9, 26), such as irregularities in the distribution of compensation to study participants. In some clinical trials, due to the nurses’ insufficient understanding of ethical risks, they failed to detect and correct the unreasonable compensation distribution to study participants in a timely manner, which triggered dissatisfaction and questioning of study participants, and harmed the reputation of the clinical trials and the rights and interests of the study participants.

Through the comparison chart in Table 2, it can be clearly seen that clinical research nurses in developed countries have more comprehensive and in-depth duties in the management of the whole cycle of trials, covering all key aspects from research design to summary of results, and excel in specialized operations and interdisciplinary collaboration. While clinical research nurses in developing countries play an important role in the basic execution work, there are obvious deficiencies in research design participation and ethical review capability. They need to further strengthen the professional capability development and expand the scope of duties in order to improve the overall quality and level of clinical trials in their countries.

**Table 2:** CRNs’ core competencies in different trial stages.

Competency	Pre-trial	During trial	Post-trial
Clinical trial regulations and ethics	Prepare ethical review materials.	Ensure protocol adherence, monitor AEs.	Assist in data cleaning and summary report writing.
Clinical research expertise	Develop study participant recruitment strategies.	Execute dosing regimen, monitor study participants.	Conduct long-term follow-up of study participants.
Communication and coordination capability	Hold patient education sessions.	Collaborate with an interdisciplinary team, communicate with study participants.	Report study findings, participate in academic exchanges.
Data management capability	Screen study participants, collect baseline data.	Record AEs, manage trial data.	Analyze and summarize data, ensure data accuracy.
Quality control capability	Review trial protocols.	Monitor protocol adherence, detect and correct violations.	Validate data, ensure trial quality.
Study participant protection capability	Lead informed consent process.	Provide nursing care, monitor safety.	Provide ongoing education and psychological support.
Teamwork capability	Coordinate with the research team.	Facilitate cross-departmental communication.	Share knowledge with team members.

It is worth noting that in certain institutions, clinical research nurses are also responsible for entering data into electronic case report forms (eCRFs) and reporting SAEs to the study sponsor. In other institutions, these tasks fall solely to the research coordinators. This could help better reflect the diversity of CRN responsibilities in practice (Table 3).

**Table 3:** Differences in the duties of clinical research nurses in developed and developing countries.

Comparison item	Clinical research nurses in developed countries	Clinical research nurses in developing countries
Whole trial cycle management—pre-phase	Participate in the preparation of ethical review materials and develop study participant recruitment strategies (e.g., holding patient education sessions to enhance enrollment efficiency).	Participate in participant screening and assist in the signing of the informed consent form.
Whole trial cycle management—mid phase	Precise execution of dosing regimen (e.g., gradient dose infusion for antitumor drugs in Phase I trial), real-time recording of AE/SAE, and activation of emergency response plan.	Distribution and recovery of trial drugs, manual entry of CRFs.
Whole trial cycle management—post phase	Lead follow-up work of study participants (e.g., long-term safety follow-up of the novel coronavirus vaccine), assist in data cleaning, and summary report writing.	Organize trial documents.
Specialized scope of operation- special techniques	Knowledge of study-specific skills such as the operation of electric pulse equipment, cold chain management of biological samples.	Less involved in special techniques.
Specialized scope of operation—interdisciplinary collaboration	Collaborate with pharmacologists and statisticians to ensure that data collection meets protocol requirements (e.g., control of time windows for pharmacokinetic specimen collection).	Less interdisciplinary collaboration, more focused on basic execution work.
Duty fulfilment gap—participation in research design	Deep participation in trial protocol design, feasibility assessment, and active participation in trial process optimization.	Low participation rate in trial process optimization (only 12%), high rate of protocol violation in phase I trials.
Duty fulfilment gap—ethical review capability	Strong ethical review capability, able to effectively identify and respond to potential ethical risks.	Weak ethical review capability, less than 20% can independently identify potential ethical risks (e.g., irregularities in the distribution of compensation to study participants).

The above data has been collated from multiple sources and is based on the comprehensive analysis of the following articles. Lin et al. (5), Chen et al. (9), Zheng et al. (26).

## 4 Exploration and analysis of the career development path of clinical research nurses in developed and developing countries

### 4.1 Mature career development model in developed countries

#### 4.1.1 Multi-level cultivation system

Clinical research nurses in developed countries have constructed a scientific and systematic cultivation system at multiple levels, covering multiple key stages, providing a clear path for nurses’ career growth. In the introductory stage, completion of GCP certification is the basic requirement, which ensures that nurses have the basic norms and operational capabilities of clinical trials. On this basis, attending special training for research nurses becomes a key link. In the US, for example, the IACRN certification course is highly recognized, which not only contains a wealth of theoretical knowledge explanation but also more than 100 h of hands-on training (27). Through these practical operations, nurses are able to practice key skills such as study participant communication, trial process execution, and data recording, in simulated and real clinical trial scenarios, laying a solid foundation for formal entry into clinical research.

With the accumulation of experience and the enhancement of professional competence, clinical research nurses enter an advanced stage and begin to develop in-depth in the direction of subspecialties. In the field of oncology research, nurses need to have an in-depth understanding of the pathogenesis of various types of tumors, therapeutic methods, and the special requirements of clinical trials. For breast cancer clinical trials, nurses not only need to master routine nursing skills, but also need to understand supportive care skills, treatment choices, and decision-making, acute care needs, managing common symptoms, as well as how to provide psychological support and rehabilitation guidance to study participants (28). A number of nurses working in the field of public health and family medicine, whose primary responsibility is to administer vaccines and provide vaccine-related education, on the other hand, need to focus on various stages of vaccine development, including immunogenicity and safety assessment of vaccines, as well as vaccination strategies for different age groups and populations (29). This stage usually requires nurses with a master’s degree and more than 5 years of extensive experience in order to cope with the complex challenges in the subspecialty area. A literature review of the role of the clinical research nurse in cancer care and analysis of published nursing practice frameworks suggests that the role of the clinical research nurse in cancer care has the potential to reach an advanced level of practice, which offers further career pathways for nurses (30).

After a long period of professional accumulation and practice precipitation, clinical research nurses are expected to reach the expert stage and play a key role in multidisciplinary teams to improve the quality and safety of patient care by sharing expertise and experience (7). At this point, with their profound professional knowledge and excellent practical ability, they take up important positions such as research head nurses and institutional GCP training instructors. As research head nurses, they are responsible for the daily management of the team, task allocation, quality control, and other tasks to ensure the smooth progress of clinical trials. As institutional GCP training instructors, they pass on their experience and professional knowledge to new nurses and other researchers to enhance the level of expertise of the entire team. At the industry level, they actively participate in the development of industry standards and contribute to the standardization of clinical research nursing. The core competency index system for clinical research nurses, developed by NIH of the US, for example, unites the wisdom and experience of clinical research nurses in many expert phases, and provides an important guidance basis for the development of the field (8). CRNs in the Italian Cooperative Oncology Research Group prepared the first nursing summary for the international trial, including strategies for staff and patient education and necessary nursing interventions (31).

#### 4.1.2 Support mechanism

The career development of clinical research nurses in developed countries cannot be separated from a perfect supporting mechanism, and a professional organization and a reasonable remuneration system are the key elements. IACRN, as a highly influential professional organization in the industry, plays a core role in the career development of clinical research nurses. IACRN provides comprehensive qualification certification services, and its certification standards are strict and scientific, covering all aspects of clinical trials, including knowledge of regulations, specialized skills, and codes of ethics. Nurses who have passed the certification are not only recognized for their professional competence, but also gain stronger competitiveness in the job market. IACRN also regularly organizes academic exchange activities, such as international symposiums, academic forums, which set up a communication platform for clinical research nurses around the world. In these activities, nurses can share the latest research results, practical experience, learn about the cutting-edge dynamics and development trends of the industry, and broaden their horizons and ideas.

A reasonable remuneration system is an important guarantee to attract and retain excellent clinical research nurses. The remuneration system of clinical research nurses in developed countries usually adopts the mode of billing according to the difficulty and workload of clinical trial projects. For some complex multi-center clinical trial projects involving multiple drugs, multiple research centers, and a large number of study participants, nurses are required to invest more time and energy, and their remuneration will be higher accordingly. According to statistics, the annual salary of senior clinical research nurses can be as high as US$100,000–US$150,000, which is about RMB 700,000–RMB 1.05 million. This kind of remuneration system fully reflects the value of nurses’ work, and motivates them to continuously improve their professional competence, undertake more challenging work tasks, and contribute to the high quality of clinical trials (32). In addition, professional academic practice research scholarships can better cultivate nurses’ participation in research (27).

### 4.2 Career development bottlenecks and exploration in developing countries

#### 4.2.1 Existing problems

Clinical research nurses in developing countries face many challenges in the process of career development, and the lack of a training system is the primary problem. Currently, only 25% of tertiary hospitals have standardized training bases for CRNs, which means that most clinical research nurses are unable to obtain systematic and professional training (10). Due to the lack of unified standards in China, the existing teaching materials and courses mostly refer to standards in developed countries, and these contents may differ from the actual national conditions and clinical practice in China, resulting in poor training effects. In some training courses, insufficient consideration has been given to factors such as China’s unique healthcare system and patients’ cultural background, making it difficult for nurses to effectively apply what they have learnt in their actual work (10). In a survey on nurses’ noncompliance with standard operating procedures (SOPs) and during clinical trials, nurses’ compliance with SOPs improved significantly after training, with 21 violations before training reduced to 3 after training (5). This reinforces the importance of building the training system.

Ambiguous promotion paths are also an important factor restricting the career development of clinical research nurses. According to the survey, 85% of CRNs are affiliated with the nursing department (11), which makes their professional title evaluation mainly dependent on the clinical nursing sequence. However, the content and focus of the work of clinical research nurses differ significantly from that of traditional clinical nursing, making it difficult for the existing professional title evaluation criteria to accurately measure their contribution and competence in the research field. This has led to clinical research nurses facing dilemmas in the process of career promotion and a lack of a clear direction for career development, which affects their motivation and sense of professional identity (12).

Low career identity is also a problem that cannot be ignored. The survey showed that 63% of CRNs felt that their own role is not fully recognized by researchers (1), which is mainly due to the lack of clear role positioning of clinical research nurses, whose unique value and professional competence in clinical trials are not fully demonstrated. In some clinical trial teams, researchers pay more attention to the opinions of doctors and other professionals and neglect the important role of clinical research nurses in study participant management and data collection, leading to a weak sense of professional belonging among clinical research nurses and confusion about their own career development (33).

#### 4.2.2 Construction of localization development path

In view of the above problems, China has actively explored localized career development paths for clinical research nurses, and promoted the professional development of clinical research nurses from the aspects of capacity building, management mode innovation, and policy support. In terms of capacity building, it is a key initiative to build a two-dimensional training system of “basic competence + research special competence.” Take the Core Competency Evaluation Index System for Clinical Research Nurses as an example, which covers seven major indicators, including clinical trial regulations and ethics, clinical research expertise, communication and coordination capability, data management capability, quality control capability, capability for protecting study participants, and teamwork capability. Through systematic training and practice, clinical research nurses can comprehensively improve their professional competence and better adapt to the needs of clinical trials (16).

The management mode innovation is also an important direction of exploration. Some medical institutions have piloted the management mode of “centralized management + specialized grouping” and achieved good results. Sun Yat-sen University Cancer Center groups CRNs according to tumor types, with each group focusing on the clinical trial research of a specific tumor type. This model enables nurses to gain an in-depth understanding of the characteristics of the tumor, treatment methods, and clinical trial requirements, and improves the quality of specialized research nursing care. Centralized management enables optimal allocation of resources, strengthens the unified management and training of clinical research nurses, and enhances the efficiency and collaboration of the entire team (34).

Policy support is crucial for the career development of clinical research nurses. Promoting the inclusion of CRN positions in the personnel configuration standards of the Pharmaceutical Preparation Certificate for Medical Institutions and clarifying the scope of practice and qualification requirements is an important manifestation of policy support. This not only improves the professional status of clinical research nurses but also provides a policy guarantee for their career development. By clarifying the scope of practice, it can avoid the overlap in responsibilities between clinical research nurses and other roles and make their work more professional and standardized. Clarifying the qualification requirements can help attract more excellent nursing talents to join the field of clinical research and enhance the professional level of the whole industry (26).

## 5 Current situation analysis and challenges

### 5.1 Root causes of differences in China and international development

#### 5.1.1 Institutional environment

Developed countries have clear and strict regulations on clinical research nurse positions in terms of the institutional environment of clinical trials. Taking the US as an example, its GCP regulations clearly mandate that research teams must be equipped with full-time clinical research nurses. This regulation provides a solid institutional foundation for the career development of clinical research nurses, making them an indispensable role in clinical trial teams. Under this institutional guarantee, clinical research nurses are able to fully dedicate themselves to clinical trial work, focusing on improving their professional competence in the field of research and providing all-around support for the smooth progress of clinical trials.

In contrast, China is currently in an exploration stage in terms of job setting standards for clinical research nurses, lacking clear regulations and institutional guidance. This has led to large differences in the provision of clinical research nurses in various medical institutions. Some hospitals have an insufficient understanding of the importance of clinical research nurses and have not set up full-time positions, making the work of clinical research nurses often replaced or neglected by other positions. In some small medical institutions, due to the lack of institutional constraints, clinical trial work is mainly performed by clinical nurses on a part-time basis, and these nurses manage heavy clinical workloads and balance clinical trial work, making it difficult to ensure the quality and efficiency of clinical trials (35).

#### 5.1.2 Education system

The nursing education system in developed countries started earlier and developed more maturely in the cultivation of clinical research nursing direction. Many nursing colleges and universities in the US, the UK, and other countries have specifically opened the direction of “clinical research nursing,” and clinical research-related courses are included in the professional core curriculum system. These courses cover clinical trial regulations, research design, data management, ethical review, and other aspects, providing students with systematic and comprehensive clinical research knowledge and skills training. Taking the Rory Meyers College of Nursing of New York University as an example, its clinical research nursing students not only have to learn basic nursing knowledge, but also have to study in-depth clinical trial management, biostatistics, and other specialized courses, and through the combination of theoretical learning and practical operation, students are cultivated to have solid clinical research ability (36). A Danish orthopedic hospital has designed educational interventions to enhance nurses’ research abilities and evidence-based practices, in order to meet their interest and intrinsic motivation for research (37).

Nursing education in China has long focused on the cultivation of clinical practice skills, and research-related courses account for a low proportion in the curriculum system. A survey of nurses in tertiary hospitals in China shows that although their research participation rate (4.13% in research projects, 7.85% in research participation, 5.35% in paper publications, 2.04% in patents) and research ability are low, they have a high demand for research training (11). However, most nursing students only study a small number of nursing research courses at the undergraduate level, and the content is mostly basic theoretical knowledge, lacking in-depth guidance on the practical operation of clinical research. In some nursing colleges and universities, nursing research courses are only used as elective courses, and students do not pay enough attention to clinical research, resulting in a lack of the necessary professional knowledge and skills when they enter the field of clinical trials after graduation, as well as having difficulty in fulfilling the duties of clinical research nurses. This difference in the education system makes the gap between clinical research nurses in China and developed countries in terms of professionalism and competence level, which restricts the career development of clinical research nurses and the improvement of the quality of clinical trials in China (25).

### 5.2 Common challenges

#### 5.2.1 Balancing study participant protection and research efficiency

In clinical trials, how to balance study participant protection and research efficiency is a key challenge common to clinical research nurses in developed and developing countries. Study participant protection is the primary principle of clinical trials, ensuring that study participants participate in the trial on a fully informed and voluntary basis, and safeguarding their safety and rights and interests from being infringed upon in the course of the trial. However, in practice, due to the complexity and urgency of clinical trials, the informed consent process is sometimes simplified in order to accelerate the trial process, which poses a potential threat to the rights and interests of study participants.

In a Phase III diabetes drug clinical trial in a hospital in China, due to the research team’s eagerness to complete the recruitment task of the trial, the clinical research nurse failed to adequately explain the potential risks and benefits of the trial to the study participants during the informed consent session, resulting in two cases of study participants unknowingly experiencing SAEs, which were omitted from the report by the research team. This incident not only compromised the health rights of the study participants but also had a serious impact on the scientificity and credibility of the trial (32).

Similarly, there are similar problems in some clinical trials in developed countries. For example, during the process of obtaining informed consent for blood transfusions, the process was sometimes rushed or incomplete, and insufficient time was given to both the doctor and patient to discuss the process, resulting in patients sometimes having an inadequate understanding of the risks and benefits of blood transfusions, and problems such as excessive psychological pressure occurred in the trial process (38).

In order to achieve a balance between study participant protection and research efficiency, clinical research nurses need to continuously optimize their workflow and improve their communication skills and professional competence in their work. In the informed consent session, plain language and diverse communication methods are used to ensure that study participants fully understand the content of the trial; in the trial process, monitoring and caring for study participants are strengthened, and AEs are detected and dealt with in a timely manner, while the trial protocols and regulatory requirements are strictly adhered to in order to ensure the authenticity and reliability of the trial data (1, 39).

#### 5.2.2 Adaptation to digital transformation

With the rapid development of information technology, the field of clinical trials is experiencing digital transformation, and digital tools such as electronic data capture (EDC) systems are increasingly used in clinical trials. Developed countries started earlier in digital transformation and have popularized EDC systems, and clinical research nurses are able to proficiently use these systems for data collection, management, and analysis, which greatly improves work efficiency and data quality.

In a clinical trial of tumor drugs in the US, clinical research nurses achieved real-time collection and transmission of study participant data through the Medidata Rave EDC system, which enabled researchers to view the latest data of study participants at any time and make timely adjustments to the treatment plan, perform real-time monitoring of data flow, patient safety, and medications (40).

Although China is also actively promoting the digital transformation of clinical trials, the current ability of clinical research nurses to apply digital tools still needs to be improved. Many clinical research nurses in China are not skilled enough in the operation of digital tools such as the Medidata Rave EDC system, which leads to a high data entry error rate and inefficient data management.

**Figure 1:**
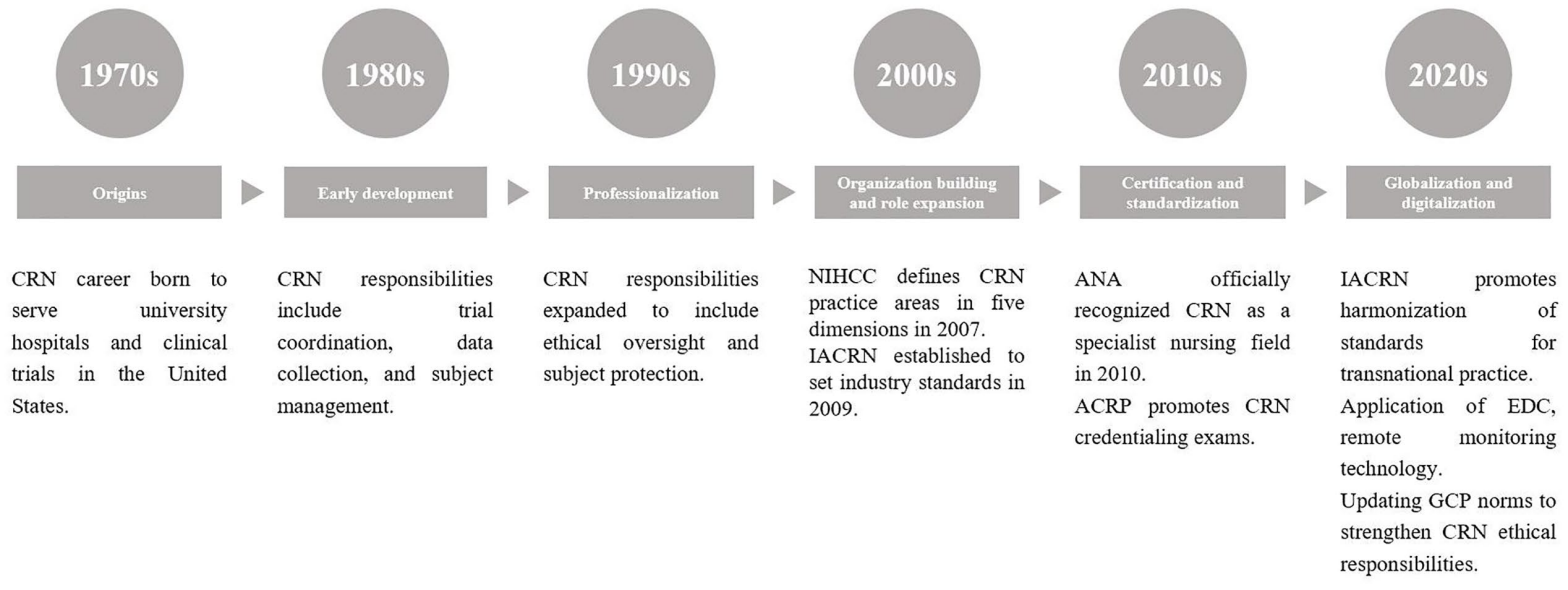
The development of CRNs in developed countries. The above milestones are compiled from the official websites of ANA, IACRN, ACRP, and NIHCC.

**Figure 2:**
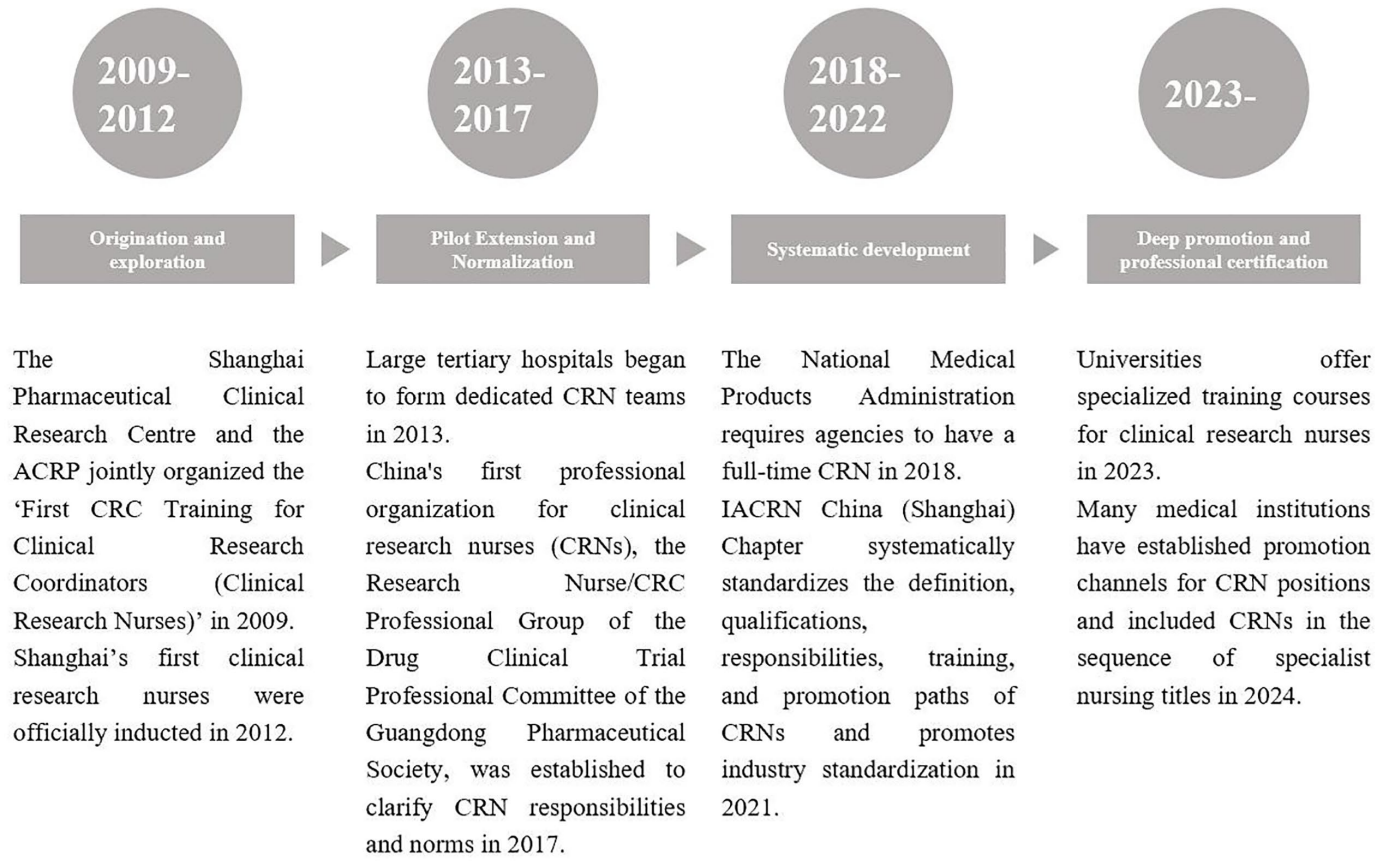
The development of CRNs in China. The above milestones are compiled from the official websites of Shanghai Pharmaceutical Clinical Research Centre, ACRP, National Medical Products Administration, IACRN China (Shanghai) Chapter, and Guangdong Pharmaceutical Society.

In order to adapt to the needs of digital transformation, clinical research nurses in China need to strengthen digital skills training and improve their ability to operate tools such as electronic data management systems and data analysis software. Medical institutions should increase their investment in digital infrastructure and provide sound training and technical support to help clinical research nurses better master digital tools and improve the digital level and quality of clinical trials (10) (Figures 1, 2).

From the above two timelines, it can be seen that CRNs in developed countries (e.g., the US) started in the 1970s, and through the establishment of organizations, standardization of accreditation, and globalization, they have formed a mature system, with responsibilities covering trial coordination, ethical supervision, and their professional status is solid. China’s CRN was introduced in 2009, and has been developing rapidly under the drive of policies: from pilot to popularization, and from coordination to research and implementation; although it started late, it is gradually narrowing the gap with the international arena through the regulatory requirements of the NMPA, the promotion of the Chinese branch of the IACRN, and the role of the IACRN in the construction of systematic training and promotion channels in colleges and universities. At present, China needs to strengthen professional certification, regulatory protection, and standardization collaboration to achieve localization and internationalization of the CRN profession.

## 6 Enlightenment on the professionalization of clinical research nurses in China

### 6.1 Policy level

#### 6.1.1 Formulation of the norms for job setting and Management of Clinical Research Nurses

The National Health Commission and other relevant departments promptly organize a team of experts to conduct an in-depth research on the current situation and trend of the development of clinical research nurse in developed and developing countries, and formulate the Norms for Job Setting and Management of Clinical Research Nurses in accordance with China’s actual national conditions. In this specification, the qualification entry conditions for clinical research nurses are clearly defined, stipulating that only those who have the qualification of registered nurses and have gone through systematic GCP training and obtained qualified certificates are qualified to work as clinical research nurses. At the same time, it defines in detail the specific duties of clinical research nurses in each stage of clinical trials, including pre-trial protocol review, study participant recruitment preparation, nursing operation, data collection and monitoring during the trial, post-trial follow-up management and data summary, to ensure that the work of clinical research nurses is regulated by rules and regulations, and that their duties are clearly defined.

#### 6.1.2 Incorporate CRN training into national continuing medical education programs

In order to enhance the professional competence and comprehensive quality of clinical research nurses, clinical research nurse training should be incorporated into the national system of continuing medical education programs. The National Health Commission should unify the planning and organize the preparation of training materials for clinical research nurses that are suitable for China’s national conditions, and the contents of the teaching materials should cover various aspects, such as clinical trial regulations, research design, ethical review, data management, and nursing operation skills. A nationally unified core competency assessment system for clinical research nurses should be established, with clearly defined assessment criteria and processes, and the content of the assessment should include theoretical knowledge, practical skills, and professionalism. Clinical research nurses can obtain corresponding credits by participating in the training of continuing medical education programs and passing the assessment, which will serve as an important basis for their professional title promotion and career development, so as to motivate clinical research nurses to continuously learn and improve their professional competence (33).

### 6.2 Institutional level

#### 6.2.1 Establishment of independent clinical research nurse positions

Each medical institution should fully recognize the important role of clinical research nurses in clinical trials and establish independent clinical research nurse positions that are managed in parallel with clinical nursing positions. Provide clinical research nurses with a specialized working space and the necessary working equipment to ensure that they can focus on clinical trials. Implement a professional title promotion channel parallel to the clinical nursing sequence, formulate professional title evaluation criteria suitable for clinical research nurses, and focus on assessing their work performance, professional competence, and scientific research achievements in clinical trials. Establish title grades for junior, intermediate, and senior clinical research nurses to provide a clear career development path for clinical research nurses and improve their sense of professional identity and belonging (16).

#### 6.2.2 Establishment of a reasonable assessment program

The performance assessment of CRN includes job competency and work performance assessment, which involves basic medical knowledge, clinical research expertise, work skills, and professional quality. Workload assessment includes the number of trials, screeners, enrollees, and visits. Work quality assessment includes the authorization error rate and the number of protocol deviations. Comprehensive assessment of CRNs through quantitative indicators can scientifically reflect their status, guide CRNs to clarify their work priorities, and provide a basis for management decisions. At the same time, it is productive for the formulation of work standardization and guarantees the quality of clinical research (23).

#### 6.2.3 Establishment of “researcher-CRN-CRC” division of labor and collaboration mechanism

Medical institutions should establish a “researcher-CRN-CRC” division of labor and collaboration mechanism, and clearly define the duties and division of labor among the three parties in clinical trials. Clearer professional identities and role boundaries should be established to facilitate the smooth progress of clinical trials (1). The researcher is mainly responsible for the design, implementation and guidance of the study protocol, and is responsible for the scientificity and results of the trial; the clinical research nurse is responsible for the nursing care of the study participants, the implementation of the trial process, and the collection and monitoring of the data, so as to ensure the safety of the study participants and the accuracy of the trial data; the CRC is responsible for the trial’s administrative coordination, document management, and communication and liaison, so as to ensure the smooth progress of the trial. Formulate SOPs, stipulate in detail the workflow and operating specifications of the three parties in all aspects of the clinical trial, strengthen communication and collaboration among the three parties, and improve the efficiency and quality of the clinical trial (34).

### 6.3 Individual level

#### 6.3.1 Actively obtain GCP certificate, ICH-GCP and other international certifications

In a survey of training requirements for clinical research nurses in Europe, 86.5% of the 37 countries surveyed required a nursing degree to become a research nurse.81.1% required completion of a GCP course, and 93.3% required regular renewal of these certifications. 67.6% reported the need for additional specialized training (41). Therefore, clinical research nurses should actively participate in GCP training, obtain a GCP certificate through the examination, and master the basic requirements and operational skills of GCP. With the international development of clinical trials, clinical research nurses should also strive to obtain international certificates such as the International Council for Harmonization of Technical Requirements for Pharmaceuticals for Human Use-Good Clinical Practice (ICH-GCP), to understand the latest regulations and standards of international clinical trials, and to improve their ability in cross-cultural study collaboration. By participating in international academic exchange activities, online learning courses, nurses can continuously update their knowledge and skills to keep pace with the development of the international clinical research nursing field.

#### 6.3.2 Deep ploughing into subspecialty fields

**Figure 3:**
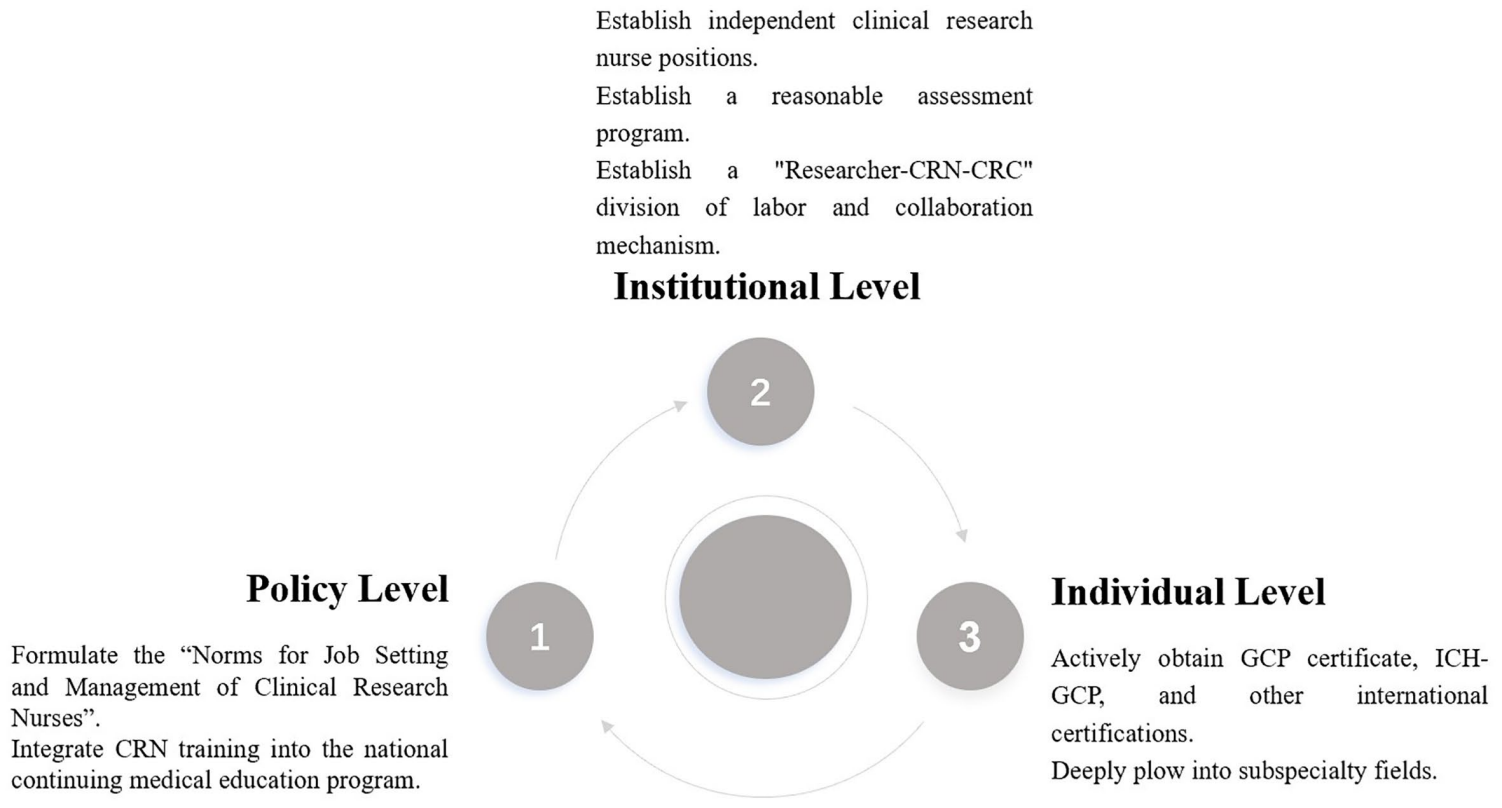
Enlightenment on the professionalization of clinical research nurses in China from policy, institutional, and individual levels.

Clinical research nurses should choose one or more subspecialty fields for deep ploughing according to their own interests and professional expertise, such as specific research scenarios in oncology, vaccine, and cardiovascular. Learn knowledge related to the Bachelor of Nursing profile, such as evidence-based practice, to enhance competence (42). Deeply study the disease knowledge, treatment methods, and clinical trial characteristics in the field and accumulate rich practical experience. Participate in clinical trial projects in the subspecialty area, work closely with experts and research teams in the area, and continuously improve professional competence and influence in the area. Actively carry out nursing research in the field of subspecialties, explore nursing models and methods suitable for this field, and provide more professional nursing support for the development of clinical trials (36) (Figure 3).

## 7 Methodology

### 7.1 Research methodology approach

#### 7.1.1 Literature review

Through systematic retrieval and screening of relevant literature, this study examined the current status and variations in the role positioning, duty comparison, and career development path of clinical research nurses in developed and developing countries. The literature review encompassed the definition of CRNs, their scope of practice, career development pathways, and the challenges and opportunities they encounter.

#### 7.1.2 Comparative method

A comparative analysis of the role positioning, duties, and career development paths of CRNs in developed countries (such as the US and the UK) and developing countries (such as China) is conducted to identify the principal differences and shared challenges between the two.

#### 7.1.3 Case illustrations drawn from the included literature

We extracted case studies from the included literature, selecting representative healthcare institutions from both developed and developing countries as subjects for in-depth analysis of their CRN management models, training systems, and career development pathways. Utilize these case studies to substantiate and supplement the findings from the literature review.

### 7.2 Data collection methods

The English-language databases utilized in this study were sourced entirely from ScienceDirect. When examining the overall developmental trajectory of clinical research nurses in developed countries, the timeframe spans 2003–2025 to ensure the research aligns with a specific historical period. When selecting representative cases from developed and developing countries for comparative analysis, the timeframe covers 2015–2025 to guarantee the research obtained is the most recent available. The Chinese-language databases utilized in this study were sourced entirely from CNKI. Given the scarcity of research in this area within China, the timeframe has been extended to cover the years 2000–2025. For both English-language and Chinese-language databases, the search strategies employed keywords or keyword combinations such as ‘Clinical research nurses’, ‘role positioning’, ‘duty comparison’, ‘career development path’, ‘developed countries’, and ‘developing countries’. Inclusion criteria comprised articles addressing topics related to the role, duties, or career development paths of clinical research nurses, published in peer-reviewed journals, in English or Chinese, and classified as reviews, empirical studies, or case studies. We conducted database searches to obtain titles and abstracts of all relevant literature for preliminary screening. Following this, we conducted a detailed screening by reading the abstracts of each article and excluding those that did not meet the inclusion criteria. Finally, we obtained the full texts of the remaining articles after preliminary screening and conducted a full-text screening to determine compliance with the inclusion criteria. The quality of the literature was ensured through an assessment of the study design, data collection and analysis methods, results and conclusions, and risk of bias.

### 7.3 Analytical methods

A systematic analysis of retrieved literature was conducted to extract key information, followed by a comparative analysis of CRNs in developed and developing countries regarding role positioning, duties, and career development paths.

### 7.4 Evaluation and justification of method selection

#### 7.4.1 The necessity of a literature review

A literature review provides the theoretical foundation and contextual information for research, ensuring its comprehensiveness and systematic approach. Through this review, gaps and shortcomings in existing research can be identified, thereby guiding the direction of subsequent studies.

#### 7.4.2 The applicability of comparative analysis

Comparative analysis facilitates the identification of differences and shared challenges in CRN management between developed and developing countries, offering insights and avenues for improvement for the latter.

#### 7.4.3 The depth of the case study

Case studies, through in-depth analysis of specific instances, can provide more concrete and vivid evidence to supplement and validate findings from literature reviews and questionnaire survey data, thereby enhancing the practicality and guidance value of this study.

#### 7.4.4 Quality control

This study conducted a narrative review with a structured literature search approach guided by the PRISMA principles, aiming to enhance transparency and reproducibility in the retrieval and screening process. However, it does not claim full adherence to all methodological requirements of a systematic review as defined by PRISMA. Data analysis involved thematic synthesis and cross-comparison of findings to support interpretive coherence, contributing to the overall credibility of the review within the scope of a narrative synthesis.

## 8 Limitations

Whilst this China-centric analysis offers valuable insights, several limitations warrant clarification in the context of cross-country comparisons.

### 8.1 Potential selection bias

This study may suffer from sample selection bias, particularly when comparing China with other countries. As the sample primarily focuses on China’s healthcare system, it may not adequately reflect the realities of other countries or regions. Caution is required when generalizing findings, implying that bias could lead to misinterpretation or overgeneralization of research outcomes.

### 8.2 Heterogeneity of developing settings

Significant variations exist among developing countries in law, funding, site governance, and workforce models. There are considerable differences in ethical review standards and implementation practices across countries, which may influence nurses’ responsibilities and roles in clinical research. Consequently, applying China’s findings directly to other developing nations requires particular attention to such environmental heterogeneity and its potential impact on interpretation.

### 8.3 The narrative nature of evidence

The qualitative data employed in this study may exhibit narrative characteristics, potentially influencing interpretations to some extent. Despite this, the study provides valuable insights through its structured approach in the literature search and screening process. Therefore, the findings should be interpreted in light of these limitations, and conclusions must be validated through data-driven verification to prevent singular narratives from dominating overall interpretations.

### 8.4 Validation and practical challenges

In China, the responsibilities and career development of clinical research nurses lack systematic standards and guidance. This results in significant variations across healthcare institutions regarding nurse staffing, functional roles, and competency requirements. The absence of balanced career planning and standards may lead to low engagement and professional development difficulties among clinical research nurses compared to standards in other countries. These institutional issues further complicate the applicability and validation of research findings.

These research limitations not only affect the interpretation of findings but also point towards directions for future refinement. Subsequent studies should consider broader multinational comparisons to mitigate selection bias and carefully analyze the specific contexts of different countries and regions to achieve more reliable conclusions. Acknowledging these limitations will foster higher-quality development within the clinical research field.

## 9 Conclusion

As a core member of the clinical trial team, the clinical research nurse’s role positioning, duty fulfilment, and career development play a key role in the quality and efficiency of clinical trials. Through an in-depth comparative analysis of clinical research nurses in developed and developing countries, we clearly recognize the gap between China and the international advanced level in this field (26).

In terms of role positioning, clinical research nurses in developed countries have a clear and independent professional identity and are deeply involved in all aspects of clinical trials, playing an indispensable role from professional advice on research design to all-around protection of the rights and interests of study participants to efficient coordination of multidisciplinary teams. On the other hand, the role of clinical research nurses in China is relatively blurred, often overlapping with the duties of clinical nurses and CRCs, and the expression of professional independence and professional value needs to be improved.

In terms of the scope of duties, clinical research nurses in developed countries undertake the duty of refined management of the whole trial cycle, from the preparation of the ethical review in the early stage, the formulation of strategies for the recruitment of study participants, to the precise administration of drugs and the monitoring of AEs during the trial, and then to the follow-up and data summary of the participants in the late stage of the study, which has formed a set of complete and efficient work system. At the same time, they are proficient in all kinds of research exclusive technologies and actively collaborate with interdisciplinary teams to provide comprehensive professional support for clinical trials. In contrast, the duties of clinical research nurses in China are mainly focused on the basic implementation level, such as study participant screening, informed consent assistance, trial drug management, and are relatively weak in research design participation, ethical review ability, and interdisciplinary collaboration.

In terms of career development path, developed countries have constructed a scientific and systematic multi-level cultivation system, which provides a clear career growth trajectory for clinical research nurses, ranging from GCP certification and specialized training at the entry stage, to in-depth development of subspecialties at the advanced stage, and then to industry leadership at the expert stage. Perfect supporting mechanisms, including qualification certification by professional organizations, academic exchanges, and a reasonable remuneration system, further motivate clinical research nurses to continuously improve their professional competencies and pursue career development. In contrast, clinical research nurses in China face many challenges, such as a lack of a training system, vague promotion channels, and low professional recognition, which limit the space and potential for their career development.

In order to enhance the professional level of clinical research nurses in China and other developing countries and promote the high-quality development of clinical trials, we need to work together at three levels: policy, institutional, and individual. At the policy level, relevant government departments should formulate the Norms for Job Setting and Management of Clinical Research Nurses as soon as possible, clarify the qualification access and job responsibilities, and incorporate CRN training into the national continuing medical education program to enhance their professional competence and comprehensive quality. At the institutional level, medical institutions should set up independent clinical research nurse positions, establish a professional title promotion channel parallel to the clinical nursing sequence, and implement an “researcher-CRN-CRC” division of labor and collaboration mechanism to improve the efficiency and quality of clinical trials (35). At the individual level, clinical research nurses should actively obtain international certifications such as the GCP certificate and ICH-GCP, continuously improve their professionalism, plough into subspecialty areas, accumulate rich practical experience, and enhance their professional influence in specific areas.

The professional development of clinical research nurses is a systematic project that requires the joint efforts of the government, medical institutions, professional organizations, and individuals. By learning from international advanced experience and combining with actual national conditions in developing countries, we will continue to explore and innovate to build a career development system for clinical research nurses, so as to inject new vitality into pharmaceutical research and development and medical research undertakings, and to promote the sustained advancement of the healthcare industry in developing countries.
